# Novel Strains of Mice Deficient for the Vesicular Acetylcholine Transporter: Insights on Transcriptional Regulation and Control of Locomotor Behavior

**DOI:** 10.1371/journal.pone.0017611

**Published:** 2011-03-10

**Authors:** Cristina Martins-Silva, Xavier De Jaeger, Monica S. Guzman, Ricardo D. F. Lima, Magda S. Santos, Christopher Kushmerick, Marcus V. Gomez, Marc G. Caron, Marco A. M. Prado, Vania F. Prado

**Affiliations:** 1 Molecular Brain Research Group, Department of Anatomy and Cell Biology and Department of Physiology and Pharmacology, Schulich School of Medicine & Dentistry, Robarts Research Institute, University of Western Ontario, London, Ontario, Canada; 2 Program in Molecular Pharmacology, School of Medicine, Federal University of Minas Gerais, Belo Horizonte, Minas Gerais, Brazil; 3 Department of Physiology and Biophysics, Federal University of Minas Gerais, Belo Horizonte, Minas Gerais, Brazil; 4 Department of Cell Biology, Duke University Medical Center, Durham, North Carolina, United States of America; Duke University, United States of America

## Abstract

Defining the contribution of acetylcholine to specific behaviors has been challenging, mainly because of the difficulty in generating suitable animal models of cholinergic dysfunction. We have recently shown that, by targeting the vesicular acetylcholine transporter (VAChT) gene, it is possible to generate genetically modified mice with cholinergic deficiency. Here we describe novel VAChT mutant lines. VAChT gene is embedded within the first intron of the choline acetyltransferase (ChAT) gene, which provides a unique arrangement and regulation for these two genes. We generated a VAChT allele that is flanked by loxP sequences and carries the resistance cassette placed in a ChAT intronic region (*FloxNeo* allele). We show that mice with the *FloxNeo* allele exhibit differential VAChT expression in distinct neuronal populations. These mice show relatively intact VAChT expression in somatomotor cholinergic neurons, but pronounced decrease in other cholinergic neurons in the brain. VAChT mutant mice present preserved neuromuscular function, but altered brain cholinergic function and are hyperactive. Genetic removal of the resistance cassette rescues VAChT expression and the hyperactivity phenotype. These results suggest that release of ACh in the brain is normally required to “turn down” neuronal circuits controlling locomotion.

## Introduction

Acetylcholine (ACh) is the major peripheral neurotransmitter controlling the parasympathetic and the sympathetic autonomic nervous system as well as the somatic motor system. Moreover, the cholinergic system is thought to play key roles in many functions in the CNS, including the control of locomotor activity, emotional behavior, and higher cognitive processes such as learning and memory [1]–[3]. Changes in cholinergic neurotransmission are associated with a variety of important neurological disorders including Alzheimer's disease, schizophrenia, Parkinson's disease, epilepsy and attention-deficit hyperactivity disorder [4].

ACh changes cellular activity of target cells through metabotropic muscarinic receptors [2], [5] and ionotropic nicotinic receptors [3], [6]. The brain expresses five different types of muscarinic receptors (M1–M5). The nicotinic receptors, which are formed by five identical or homologous subunits, are generated from twelve different subunits (nine α-subunits and three β-subunits) [3]. The various pentameric nAChR subunit combinations have different pharmacological and kinetic properties, and are widely distributed in the brain. Similar complexity is observed for the different G-coupled muscarinic receptors. Knowledge of the interplay between different receptors is not fully understood, and because of this complexity, defining the actual contribution of brain ACh to specific behaviors has been challenging.

There have been several attempts to generate animal models of cholinergic dysfunction by elimination of cholinergic neurons using electrolytic or excitotoxic methods, which are nonselective and destroy indistinctly both noncholinergic and cholinergic neurons, as well by the more selective strategy of cholinergic immunolesion, which preferentially destroy cholinergic neurons [1]. Although these studies have provided important information regarding the cholinergic system, they also have raised a number of inconsistent results concerning behavioral processes that are affected by altering cholinergic transmission [1]. The fact that some of these techniques may not be specific and can eliminate non-cholinergic neurons or that they may not eliminate all cholinergic neurons could explain some of the differences. In addition, other signalling molecules, such as neuropeptides, growth factors and co-transmitters, can be co-released by cholinergic neurons, further confounding the interpretation of neuronal degeneration-induced cholinergic deficiency. Furthermore, neuronal death causes inflammation which can also complicate interpretation of the experiments [7]–[9]. Therefore it is important to develop alternative, more consistent and targeted approaches to complement these previous studies and to investigate specific roles of ACh in brain functions.

Using genetics to generate mouse models of cholinergic deficiency is equally challenging. ChAT KO mice die shortly after birth and adult heterozygous ChAT KO mice exhibit compensatory increases in choline uptake and show no behavioral phenotype [10], [11]. We have recently generated novel mouse lines of cholinergic deficiency by targeting the vesicular acetylcholine transporter (VAChT knockdown - VAChT KD and VAChT knockout - VAChT ^del/del^). VAChT is essential for ACh release as mice null for VAChT expression do not survive [12]. In contrast, mice with reduction of VAChT expression by 40% (VAChT KD^HET^) and 70% (VAChT KD^HOM^) are viable [13]. Analysis of ACh release in VAChT KD mice indicate that decreased expression of VAChT perturbs storage of ACh in vesicles. During stimulation, impaired ACh storage becomes more pronounced leading to significant decrease in ACh release [13], [14]. VAChT KD^HOM^ mice are myasthenic and present social and object recognition memory deficits [13] and cardiac dysfunction [15], indicating that perturbation of ACh storage affects several physiological functions [12], [13], [15]–[17]. All these phenotypes can be rescued by inhibition of cholinesterase, indicating that they are the result of decreased ACh release due to the exocytosis of partially-filled synaptic vesicles and are not the result of developmental changes [13]–[15].

The organization of the VAChT gene locus is complex. The entire VAChT open reading frame is encoded by one single exon that is contained inside the first intron of the ChAT gene [18]. This nested gene structure is frequently named cholinergic gene locus (CGL). Control of expression of VAChT and ChAT is poorly understood, and distinct cholinergic neurons show different requirements for regulatory regions within the cholinergic gene locus [19]–[22]. To further investigate the roles of the cholinergic system we have developed novel strains of VAChT targeted-mice. Our strategy was to generate a VAChT allele that is flanked by loxP sequences and carries a TK-Neo resistance cassette approximately 1.5kb downstream from the VAChT stop codon, in a ChAT intronic region. We show that interrupting the intron between ChAT exons N and M with a TK-Neo cassette maintains VAChT expression in the somatomotor subset of cholinergic neurons relatively intact, but causes a pronounced decrease in VAChT expression in other groups of cholinergic neurons in the CNS. As a consequence, these mice present preserved neuromuscular function, but altered brain cholinergic activity. We show that these new mutant mice are hyperactive when exposed to a new environment. Interestingly, hyperactivity is a behaviour trait found in several diseases such as Alzheimer's disease [23]–[25], schizophrenia [26], [27] and Attention-deficit hyperactivity disorder [28], [29]. Genetic removal of the TK-Neo resistance cassette rescues VAChT expression and the hyperactivity phenotype. These results suggest that release of ACh is normally required to “turn down” neuronal circuits controlling locomotion.

## Results

### Generation of VAChT-deficient mice

**Figure 1:**
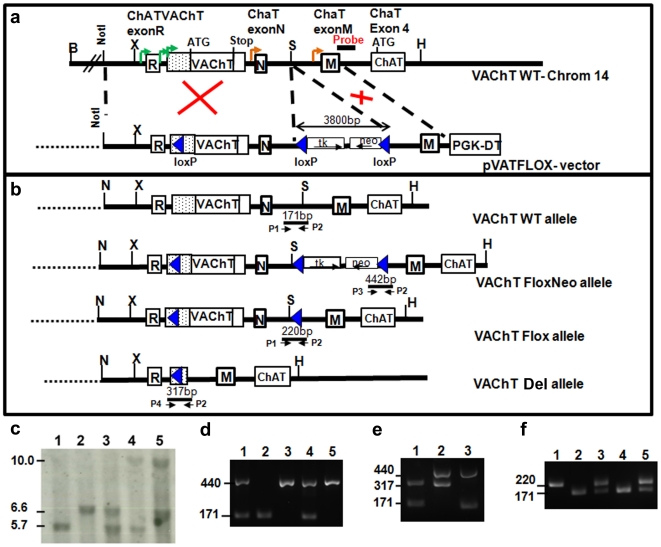
Schematic Drawing of the Cholinergic Gene Locus and Generation of VAChT Deficient Mice. a) Boxes represent the different exons of ChAT or VAChT. The position of the initiation codon (ATG) for VAChT and ChAT and the stop codon (Stop) of VAChT are indicated. Potential transcription initiation sites are indicated for VAChT (green arrowheads) and ChAT (orange arrowheads). Note that the VAChT gene is within the first intron of ChAT. b) Different VAChT alleles generated. P1, P2, P3 and P4 indicate the primers used for PCR genotyping and the fragment sizes generated. LoxP sequence, some restriction enzymatic sites and probe annealing are represented. c) Southern blot analysis of WT (lane 1), VAChT^FloxNeo/FloxNeo^(lane 2), VAChT^WT/FloxNeo^ (lane 3), VAChT^WT/Del^(lane 4) and VAChT^FloxNeo/Del^(lane 5). d) PCR analysis of VAChT^WT/FloxNeo^ (lanes 1 and 4), VAChT^WT/WT^ (lane 2), and VAChT^FloxNeo/FloxNeo^ (lanes 3 and 5). e) PCR analysis of VAChT^ WT/del^ mice (lane 1), VAChT^ FloxNeo/Del^ (lane 2), and VAChT^ WT/FloxNeo^ mice (lane 3). f) PCR analysis of VAChT^ Flox/Flox^ mice (lane1), VAChT^ WTWT^ mice (lane2 and 4), and VAChT^ WT/Flox^ (lane 3 and 5).

We generated a new VAChT targeted mouse line by inserting a lox-P flanked TK-Neo cassette in the 3′ region of the VAChT gene, in the intron between exons N and M of the ChAT gene, and a third lox-P sequence 260 bp upstream from the VAChT translational initiation codon (Figure 1). Successful recombination of the mutated VAChT allele was confirmed by Southern-blot and PCR analyses (Fig. 1C and 1D).

**Figure 2:**
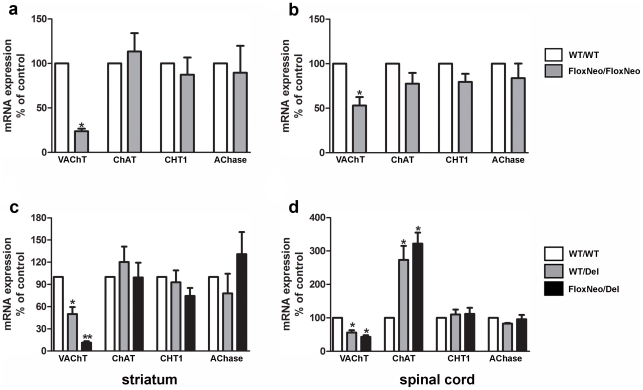
VAChT mRNA expression is changed in VAChT mutant mice. a) VAChT, ChAT, CHT1 and AChase mRNA levels in striatum and b) spinal cord of WT and VAChT^FloxNeo/FloxNeo^ mice. c) VAChT, ChAT, CHT1 and AChase mRNA levels in striatum and d) spinal cord of VAChT^WT/WT^, VAChT^FloxNeo/Del^ and VAChT^WT/Del^ mice. mRNA expression levels were quantified by qPCR using actin to normalize the data. Graphs represent average of 4–6 different mice. (*) and (**) indicate p<0.01 and p<0.001 respectively.

We initially characterized this novel mouse line by evaluating VAChT expression expecting that the new location chosen for the insertion of the TK-Neo cassette would not alter VAChT gene expression. However, we found that VAChT^FloxNeo/FloxNeo^ mice showed a large decrease in VAChT expression in the striatum (76% decrease in VAChT mRNA- Figure 2A), but VAChT expression in the spinal cord was decreased only by 46% (Figure 2B). We have found in previous experiments that decreased expression of VAChT up to 50% in the spinal cord does not alter neuromuscular function [12], [13]. As the VAChT^FloxNeo^ allele showed much pronounced decrease of VAChT expression in the brain compared to the spinal cord, it offered the chance to knock-down VAChT expression in the brain, but preserve peripheral cholinergic function. To examine this possibility, we crossed VAChT^FloxNeo/FloxNeo^ mice to heterozygous VAChT-null mice in order to generated VAChT^FloxNeo/del^ mice anticipating that this novel mouse line might present even more significant knockdown of VAChT in the brain, but relatively preserved peripheral function. Genotyping of these mice was obtained by PCR (Fig. 1E).

We examined VAChT expression in VAChT^FloxNeo/del^ mice compared to VAChT^wt/wt^ mice. The levels of mRNA for VAChT were decreased in the striatum of VAChT^FloxNeo/del^ mice even further (89% decrease - Fig. 2C). Similarly to VAChT^FloxNeo/FloxNeo^ mice, VAChT expression in the spinal cord of VAChT^FloxNeo/del^ mice was relatively preserved (57% decrease, Figure 2D). Confirming results obtained previously, levels of mRNA for VAChT^wt/del^ mice decreased 50% when compared to VAChT^wt/wt^ mice (Figure 2C and [12]). We also examined other components of cholinergic nerve terminals that can impact cholinergic tone. Of significant interest both VAChT^FloxNeo/del^ and VAChT^wt/del^ presented an increase in ChAT mRNA expression in the spinal cord, but not in the striatum (Fig. 2C and 2D) and this was compatible with previous findings for the *del* allele [12]. This increase in ChAT expression is likely related to the removal of the VAChT gene with a decrease in the distance between two ChAT promoters (see Fig. 1). In contrast, ChAT mRNA expression was not changed in VAChT^FloxNeo/FloxNeo^ mice (Fig, 2A and 2B). Also, CHT1 and AChase mRNA expression were not changed in any of the VAChT mutants in either the striatum or spinal cord (Fig. 2A–D).

**Figure 3:**
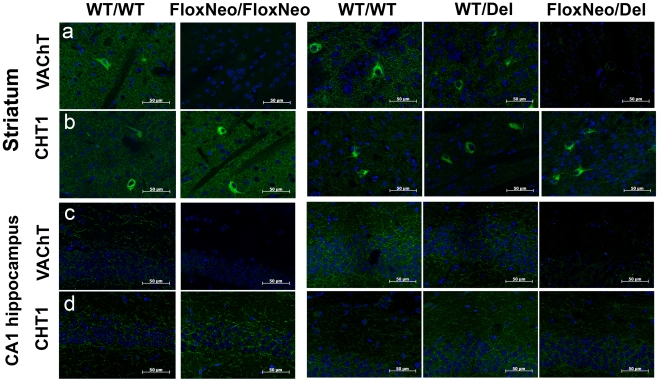
VAChT immunorreactivity is altered in VAChT mutant mice. a) Representative optical sections from striatum stained with a VAChT antibody (green) or b) stained with CHT1 antibody (green). c) Representative optical sections from hippocampus stained with a VAChT antibody or d) CHT1 antibody (green). Dapi labelling (blue) was used to stain nuclei. Scale bar 50 µm.

**Figure 4:**
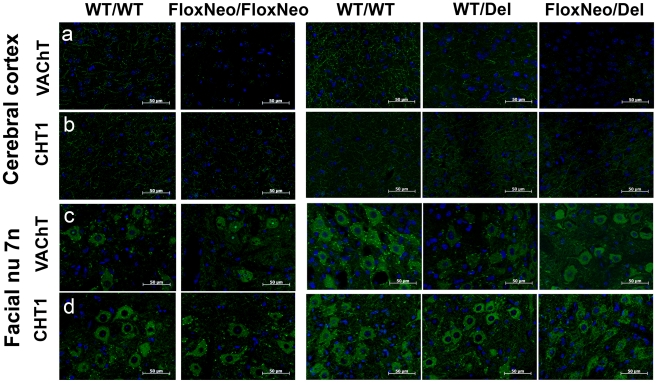
VAChT immunorreactivity is altered in VAChT mutant mice. a) Representative optical sections from cortex stained with a VAChT antibody (green) or b) CHT1 antibody (green). c) Representative optical sections from facial motor nuclei stained with a VAChT antibody or d) CHT1 antibody (green). Dapi labelling (blue) was used to stain nuclei. Scale bar 50 µm.

To investigate the expression of VAChT in distinct brain regions we used immunofluorescence. In brain sections VAChT expression was drastically reduced in the striatum, cortex and hippocampus of VAChT^FloxNeo/del^ and VAChT^FloxNeo/FloxNeo^ mice compared to VAChT^wt/del^ or VAChT^wt/wt^ mice (Fig. 3 and 4). In contrast CHT1 labelling was preserved. We also examined expression of VAChT in the facial motor nuclei (Fig. 4). Staining in the cell bodies was similar in all genotypes, although both VAChT^FloxNeo/del^ and VAChT^FloxNeo/FloxNeo^ mice had a decreased labelling in the punctated fluorescence for nerve terminals that contact these neurons. To further investigate if indeed VAChT expression was reduced in the brain, we used immunoblot analysis of striatum tissues. A decrease of 75 to 85% in the expression of VAChT in the striatum of VAChT mutants was observed (supplementary Fig. S1).

**Figure 5:**
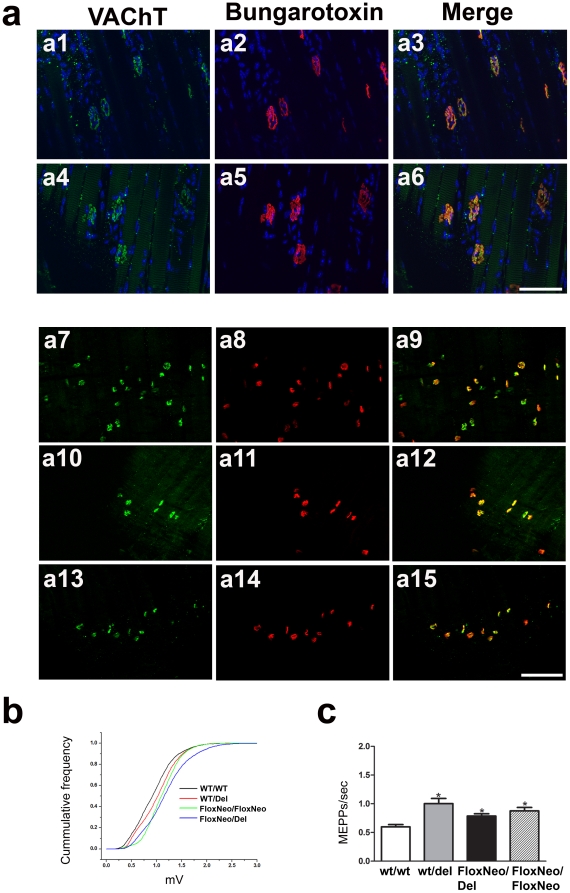
NMJ morphology and transmission in VAChT mutant mice. a) Diaphragms were immunolabelled with VAChT antibody (green) and α-bungarotoxin (red) to label nicotinic receptors. Right columns show overlay pictures. Dapi (blue) was used to stain nuclei. Images are representative of 3 independent experiments. WT control mice (a.1–3), VAChT^FloxNeo/FloxNeo^ mice (a.4–6), WT control mice (a.7–9), VAChT^WT/Del^ mice (a.10–12), and VAChT^FloxNeo/Del^ mice (a.13–15). No alterations were observed between the genotypes. Scale bar 50µm. b) Quantal size of the four genotypes quantified by plotting the cumulative frequency of MEPP amplitudes. WT control (black line), VAChT^WT/Del^ mice (red line), VAChT^FloxNeo/FloxNeo^ mice (blue line) and VAChT^FloxNeo/Del^ mice (green line). c) Frequency of MEPPs at synapses for the four genotypes. (*) indicates statistically significant difference from control wild-type mice (two-way ANOVA followed by Bonferroni post hoc; F(2,14) = 21,98, p<0.005).

To further explore VAChT expression in the periphery we stained VAChT in the NMJ of diaphragm. In contrast to the decreased VAChT expression in distinct brain regions, we found negligible differences for VAChT expression in nerve-endings at the diaphragm of VAChT^FloxNeo/del^ and VAChT^FloxNeo/FloxNeo^ mice when compared to the two control genotypes (Fig. 5A). Furthermore, analysis of nicotinic ACh receptor labelling using fluorescent bungarotoxin suggested normal nAChR distribution (Fig. 5A).

To test if neuromuscular transmission was preserved in VAChT^FloxNeo/del^ and VAChT^FloxNeo/FloxNeo^ mice we recorded from the NMJ of the diaphragm. Both the amplitude and the frequency of miniature end-plate potentials (MEPPs) were increased for VAChT^FloxNeo/del^, VAChT^FloxNeo/FloxNeo^ and VAChT^wt/del^ mice when compared to VAChT^wt/wt^ (Fig. 5B and C). These results are compatible with our previous observations that close to 50% reduction of VAChT at neuromuscular junctions affects quantal release of ACh only mildly [13]. These results suggest that quantal release in the two mutant mice with decreased expression of VAChT in the brain was well preserved at the NMJ.

**Figure 6:**
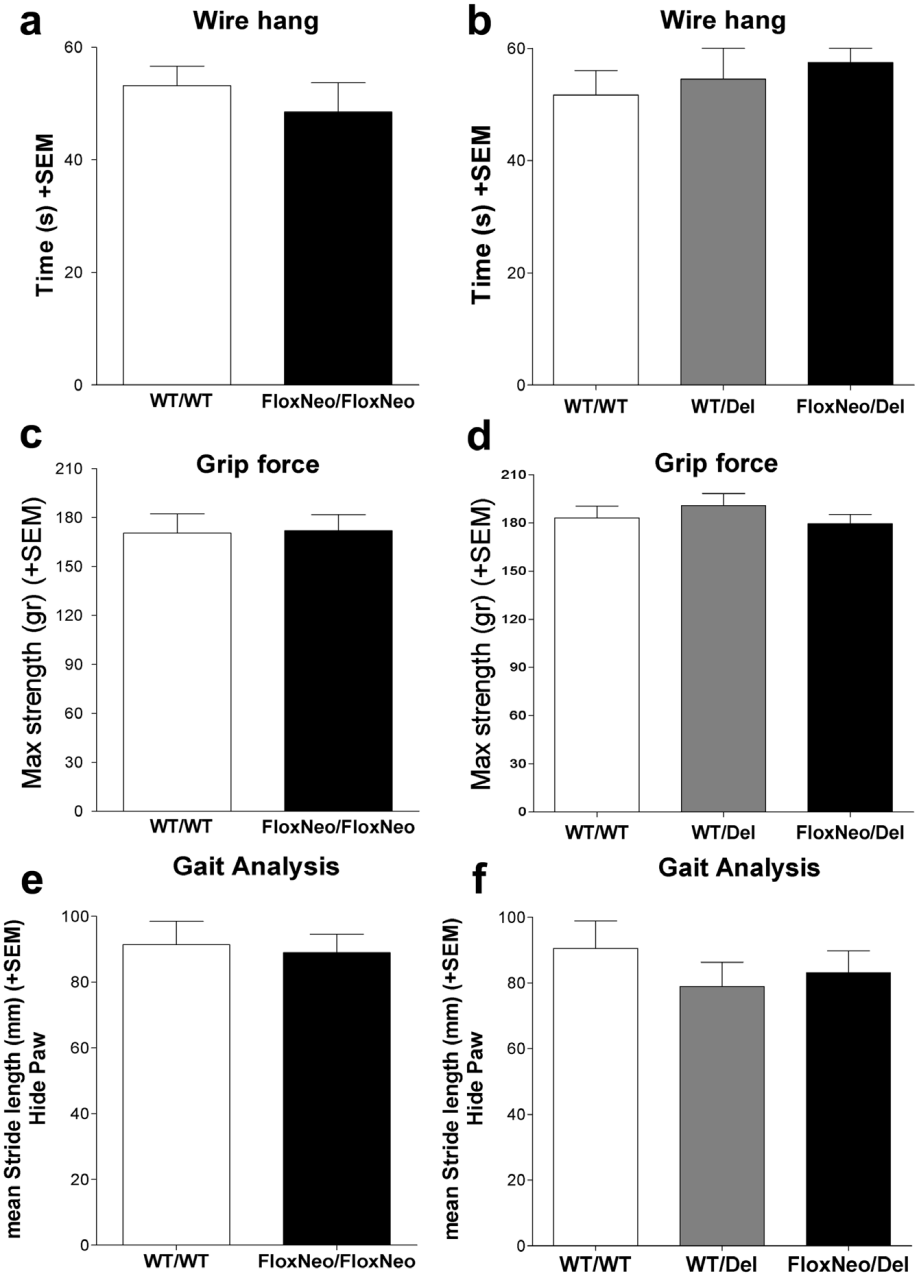
Neuromuscular function in VAChT mutant mice. a) Time spent hanging upside down from a wire netting for WT and VAChT^FloxNeo/FloxNeo^ mice. No significant difference was observed [T_(33)_ = 295, P = 0.728]. b) Wire Hang for WT, VAChT^WT/Del^ and VAChT^FloxNeo/Del^ mice. No significant difference was observed [Kruskal-Wallis, H_(2)_ = 2.604, P = 0.272]. c) Grip force measured for WT and VAChT^FloxNeo/FloxNeo^ mice. There is no significant difference between the two genotypes [T_(21)_ = 125, P = 0.689]. d) Maximal force expressed in gram. No difference was observed between WT, VAChT^WT/Del^ and VAChT^FloxNeo/Del^ mice [One way ANOVA, F_(2)_ = 0.600, P = 0. 507]. e) Gait analysis for WT, VAChT^FloxNeo/FloxNeo^ mice. No significant difference between genotypes was revealed [Student test, t_(13)_ = 0.263 P = 0. 797] f) Gait analysis for WT, VAChT^WT/Del^ and VAChT^FloxNeo/Del^. No significant difference between genotypes was observed [One way ANOVA, F_(2)_ = 0.699, P = 0. 559].

To examine whether VAChT^FloxNeo/del^ and VAChT^FloxNeo/FloxNeo^ mice had preserved muscular function we performed a series of neuromuscular tests. These mutant mice showed no difference in grip-force and wire-hang tasks suggesting preserved neuromuscular function (Fig. 6A–D). Moreover, because previous observations showed that VAChT KD^HOM^ mice had gait problems [13], we also tested if VAChT^FloxNeo/del^, VAChT^FloxNeo/FloxNeo^ and VAChT^wt/del^ mice presented any gait abnormality. In agreement with the lack of neuromuscular phenotype, we found no gait deficiency in these mutant mice (Fig. 6 E–F). These results indicate that VAChT^FloxNeo/del^ and VAChT^FloxNeo/FloxNeo^ mice, unlike VAChT-KD^HOM^ mice, do not present a neuromuscular phenotype.

**Figure 7:**
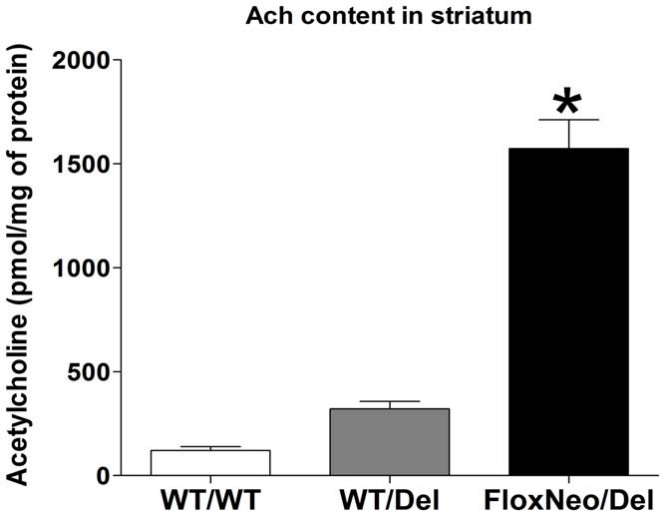
Acetylcholine content in the striatum VAChT mutant mice. Striatal tissue ACh levels for WT, VAChT^WT/Del^ and VAChT^FloxNeo/Del^ mice were assayed by chemiluminescent detection. Data represent 4–9 experiments (mean ± SEM). (One-way Anova with Bonferroni post hoc, F_(2,14)_ = 21,98, (*)p<0. 05* for wild-type controls.

We have previously demonstrated that decreased VAChT expression leads to proportional increase in the amount of total ACh in the brains of mutant mice, as ACh that is not released accumulates in nerve terminals [12], [13]. Because we did not detect any alteration in either ChAT or CHT1 in the striatum, we measured the amount of ACh in the brains of mutant mice as an indirect assessment of ACh output. We determined the ACh content in the striatum of VAChT^FloxNeo/del^ mice (the line with largest decrease in VAChT expression) and VAChT^wt/del^ mice. VAChT^FloxNeo/del^ mice presented several-fold more ACh in the striatum than wild-type controls, whereas the increase in VAChT^wt/del^ mice was around two-fold (Fig. 7). These data show a gene-dosage effect in the ACh content in these VAChT mutant mice and corroborate the mRNA and protein findings that the decrease in VAChT expression is more accentuated in VAChT^FloxNeo/del^ when compared to VAChT^WT/del^ mice.

### VAChT^FloxNeo/del^ and VAChT^FloxNeo/FloxNeo^ mice are hyperactive

**Figure 8:**
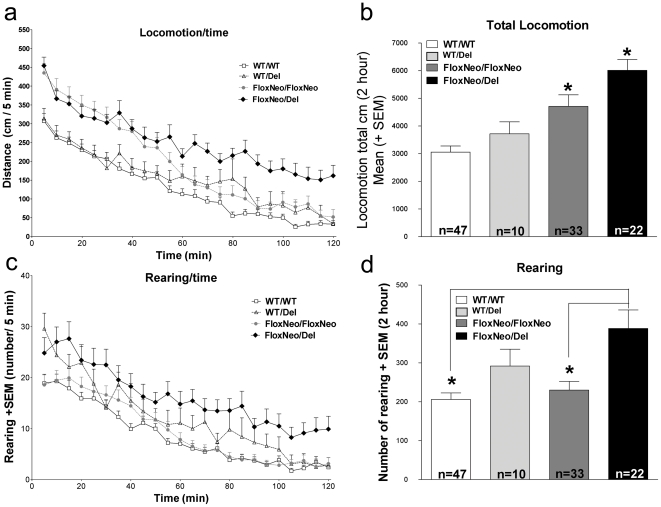
VAChT mutant mice are hyperactive. a) Spontaneous horizontal activity during two hours in the open field for WT, VAChT^WT/Del^, VAChT^FloxNeo/FloxNeo^ and VAChT^FloxNeo/Del^ mice. b) Total spontaneous horizontal activity during the two hour was increased for VAChT^FloxNeo/FloxNeo^ and VAChT^FloxNeo/Del^ mice compared to WT/WT. But no difference between WT and VAChT^WT/Del^ was observed. c) Spontaneous vertical activity during two hours in the open field for WT, VAChT^WT/Del^, VAChT^FloxNeo/FloxNeo^ and VAChT^FloxNeo/Del^ mice. d) Total number of rearings during the two hour period. Rearings for VAChT^FloxNeo/Del^ were significantly higher when compared to WT (Kruskal-Wallis test; (H_(3)_ = 13.764, post-hoc Dunn p<0.05). (*) indicate p<0.01.

As these new VAChT mutant mice have preserved peripheral function, they became candidates to explore phenotypes that were previously difficult to study using VAChT KD^HOM^ mice due to their neuromuscular deficiency. To start assessing the consequences of decreased VAChT expression for central functions we examined locomotor activity in the open field, which has been shown to be altered by antagonists of muscarinic receptors as well as by genetic elimination of some nicotinic and muscarinic receptors [29]–[37]. Figure 8 indicates that VAChT^FloxNeo/del^ mice showed increased locomotion throughout the 2 hour monitoring period when compared to wild-type controls (Kruskal-Wallis test show difference between the genotypes (H_(3)_ = 31.680, p<0.001). The average total distance traveled by VAChT^FloxNeo/del^ mice in 2 h was 2.1-fold higher than that of WT controls. An intermediate increase (1.4-fold) in locomotor activity was observed in VAChT^FloxNeo/FloxNeo^ mice when compared to wild-type controls (Fig. 8A and B). Activity levels of VAChT^wt/del^ mice showed a tendency to increase however it did not meet statistical significance, similar to previously reported observations [12]. These results suggest that decreased VAChT expression to the levels found in VAChT^FloxNeo/del^ mice causes abnormal motor activity. In addition, vertical exploration in the open field was increased in VAChT^FloxNeo/del^ mice as shown by the number of rearings (Figure 8C and D; Kruskal-Wallis test; (H_(3)_ = 13.764, p<0.05; post-hoc Dunn reveal a significant higher rearing number of VAChT^FloxNeo/del^ compared to WT controls).

**Figure 9:**
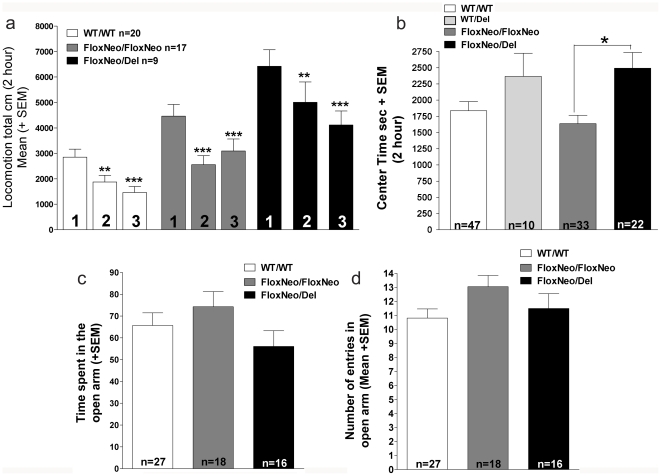
Habituation and anxiety are not changed in VAChT mutant mice. a) Habituation to open field during 3 consecutive days for WT, VAChT^FloxNeo/FloxNeo^ and VAChT^FloxNeo/Del^ mice. Mice showed no impairment in habituation in the novel environment. Two-way repeated measures ANOVA- main effect of genotype,*F*_(2, 86)_ = 15.825, *p*<0.001, day *F*_(2, 86)_ = 35.318, *p*<0.001 and interaction genotype x day *F*_(4, 86)_ = 2.505, *p*<0.05]. b) Time spend in the centre during the 2 hour in the open field for WT, VAChT^WT/Del^, VAChT^FloxNeo/FloxNeo^ and VAChT^FloxNeo/Del^ mice. VAChT^FloxNeo/del^ mice spent significant more time in the center of the open field apparatus (Kruskal-Wallis test, H_(3)_ = 11.537, p<0.05; post-hoc Dunn's method, *p*<0.05). c) Time spend in the open arm of elevated plus maze for WT, VAChT^FloxNeo/FloxNeo^ and VAChT^FloxNeo/Del^ mice was not significantly affected. One Way Analysis of Variance F_(2,58)_ = 1,603, NS). d) Number of entries in the open arm of elevated plus maze for WT, VAChT^FloxNeo/FloxNeo^ and VAChT^FloxNeo/Del^ mice was not significantly affected. One Way Analysis of Variance (F_(2,58)_ = 1,845, NS). (*), (**) and (***) indicate p<0.05, p<0.01 and p<0.001 respectively.

Lack of habituation does not seem to be the cause of the hyperactivity as all three mutants showed decreased motor activity across the 2-hour test session (Figure 8A). Moreover, VAChT^FloxNeo/FloxNeo^ and VAChT^FloxNeo/del^ mice were retested after 24 h and 48 h under the same conditions to investigate intersession habituation and both genotypes showed significant decrease in locomotor activity in the second and third days [*between-sessions habituation* in the open field; Figure 9A, two-way repeated measures ANOVA- main effect of genotype,*F*_(2, 86)_ = 15.825, *p*<0.001, day *F*_(2, 86)_ = 35.318, *p*<0.001 and interaction genotype x day *F*_(4, 86)_ = 2.505, *p*<0.05], further suggesting no impairment in habituation in the novel environment. It is important to note that even after the third day, both VAChT^FloxNeo/FloxNeo^ and VAChT^FloxNeo/del^ mice remained hyperactive when compared to WT (Tukey test respectively P<0.01 and P<0.001).

We also tested for changes in anxiety level. The time spent in the center vs. the periphery of the open field was evaluated in the same open field trials used to quantify locomotor movement. As shown in Figure 9B VAChT^FloxNeo/del^ mice spent significantly more time in the center of the open field apparatus (Kruskal-Wallis test, H_(3)_ = 11.537, p<0.05; post-hoc Dunn's method, *p*<0.05) which could be an indication of reduced anxiety [38]. However, when we assessed the willingness of VAChT mutant mice to explore a novel unprotected environment (open arms) of the elevated plus maze, the time spent in the open arms (Fig. 9C; One Way Analysis of Variance F_(2,58)_ = 1,603, NS), and the number of entries in the open arms in the elevated plus maze test (Fig. 9D; One Way Analysis of Variance F_(2,58)_ = 1,845, NS) were not significantly affected in VAChT^FloxNeo/del^ mice. These data indicate that VAChT^FloxNeo/del^ mice do not show consistent changes in anxiety-related behaviors.

### Genetic rescue of VAChT-mutant mice hyperactive behavior

If decreased VAChT expression causes hyperactivity, it would be expected that correcting VAChT levels should allow for rescue of this phenotype. The VAChT^FloxNeo^ allele carries a TK-Neo cassette 3′ from the ORF of VAChT and this is likely the cause of decreased VAChT expression (Fig. 1). Cre excision of loxP flanked DNA sequences is a stochastic event [39], we therefore crossed VAChT^FloxNeo/wt^ mice to distinct Cre mice (see Methods) to obtain an allele in which the TK-Neo cassette was deleted (Fig. 1B; VAChT^Flox^ allele). We screened the offspring from this cross by PCR to identify founder mice carrying only the floxed VAChT gene, with removal of the TK-Neo cassette. VAChT floxed founders (VAChT^Flox^) were crossed to C57BL/6J mice to confirm germ-line transmission and the progeny obtained were intercrossed to obtain VAChT^Flox/Flox^ mice and WT controls (PCR in Fig. 1F).

**Figure 10:**
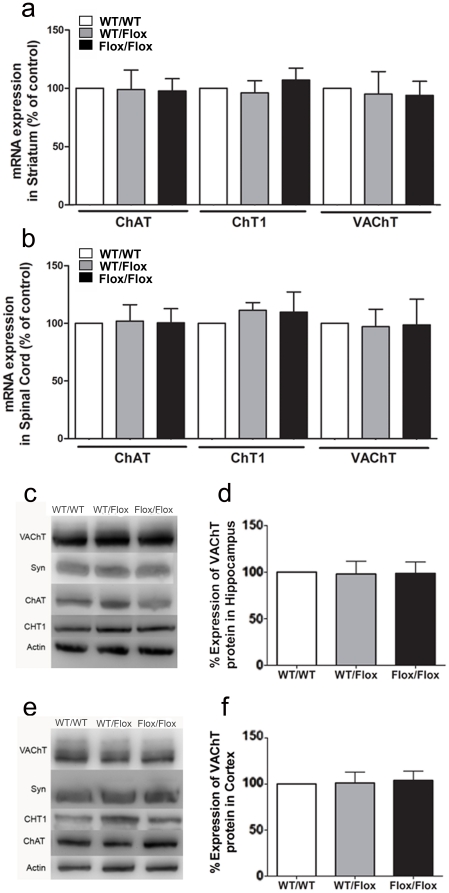
Genetic rescue of VAChT-mutant mice. a) VAChT, ChAT, and CHT1 mRNA levels were measured by qPCR in the striatum of WT mice (white bar), VAChT^WT/Flox^ (grey bar) and VAChT^Flox/Flox^ (black bar) mice. b) VAChT, ChAT, CHT1 mRNA levels in the spinal cord of WT mice (white bar), VAChT^WT/Flox^ (grey bar) and VAChT^Flox/Flox^ (black bar) mice. c) Representative immunoblot of control, VAChT^WT/Flox^ and VAChT^Flox/Flox^ mice in striatum. d) Quantification of protein levels. Actin immunoreactivity was used to correct for protein loading between experiments. Data are presented as a percentage of wild-type levels. e) Representative immunoblot of control, VAChT^WT/Flox^ and VAChT^Flox/Flox^ mice in spinal cord. f) Quantification of protein levels. Actin immunoreactivity was used to correct for protein loading between experiments. Data are presented as a percentage of wild-type levels.

**Figure 11:**
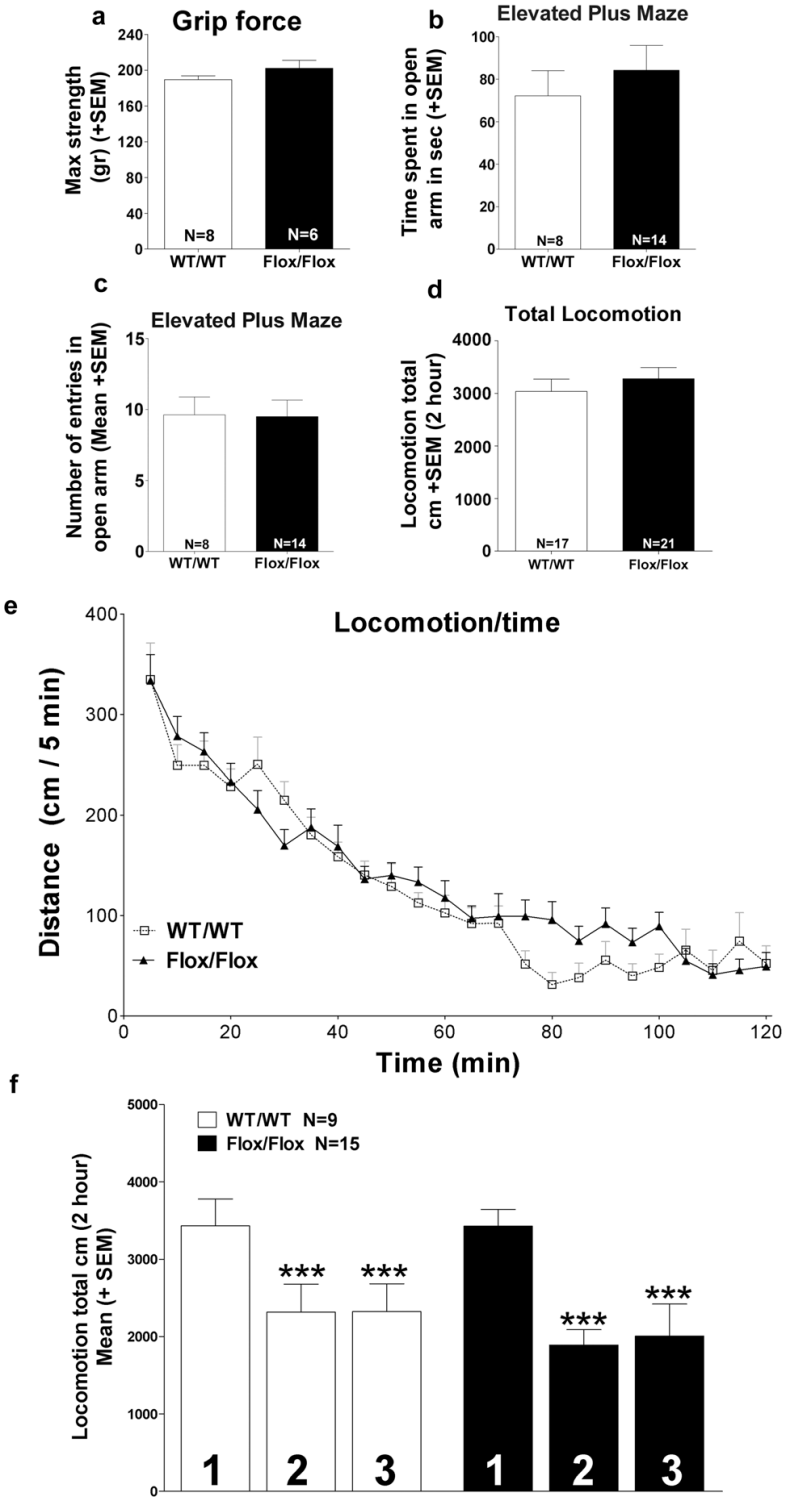
Restoration of normal phenotype by removing of the Neo-cassette. a) Spontaneous horizontal activity during two hours in the open field for VAChT^Flox/Flox^ mice. The total locomotion is similar in both genotype (t_(36)_ = −0.769 P = 0.447). b) Grip force for VAChT^Flox/Flox^ mice. (t_(12)_ =  −1.414 P = 0.183) c) Time spent in the open arm of elevated plus maze for VAChT^Flox/Flox^ mice. No difference in anxiety level was observed (t _(20)_ = −0,670, P = 0,510). d) Number of entries in the open arm of elevated plus maze for WT and VAChT^Flox/Flox^ mice. e) Spontaneous horizontal activity during two hours in the open field for VAChT^Flox/Flox^ mice. f) Habituation to open field during 3 consecutive days. The ANOVA reveal no effect of genotype (F_(1,44)_ = 0.475, P = 0.498), a significant effect of the factor day (F_(2,44)_ = 16.733, P<0.001) and no interaction genotype x day (F_(2,44)_ = 0.364, P = 0.697). Post-hoc showed difference between the Day1 and Day2, 3. (***) indicate p<0.001.

We investigated VAChT expression at the mRNA and protein levels and found that VAChT^Flox/Flox^ mice have essentially the same level of expression for this transporter as VAChT^wt/wt^ mice in the striatum, cortex, spinal cord and hippocampus (Fig. 10). Moreover, ChAT and CHT1 expression were not changed in VAChT^Flox/Flox^ mice (Fig. 10). Accordingly VAChT^Flox/Flox^ mice showed no deficits in neuromuscular function in the grip-force (Fig. 11A) or wire-hang (not shown). Measures of anxiety in the elevated plus maze were identical to measures of WT controls (Fig. 11B and C). When we tested VAChT^Flox/Flox^ mice in the open-field we also found that locomotor activity was identical to that of VAChT^wt/wt^ mice and no habituation deficits were observed (Fig. 11D–F). These results strongly suggest the recovery of VAChT expression by removal of the TK-Neo cassette rescued the hyperactivity phenotype of VAChT^FloxNeo^.

## Discussion

### The VAChT^FloxNeo^ allele shows differential regulation of VAChT expression

The present experiments explore some of the remarkable features of the cholinergic gene locus to target VAChT and generate mice with decreased cholinergic function. We show that interference with the VAChT-ChAT locus, by insertion of a TK-Neo cassette in the intron between ChAT exons N and M, differentially affected the expression of VAChT in the brain and the spinal cord. Owing to the relative preservation of cholinergic function in the spinal cord and NMJ, we were able to show that one of the consequences of reduction of VAChT expression in the forebrain, and consequent reduction of ACh release, is hyperactivity. This phenotype shows a gene-dose effect with lesser expression of VAChT causing a more pronounced hyperactivity.

VAChT has a unique genomic organization; its open reading frame is encoded within the first intron of the ChAT gene. This arrangement [18] is conserved in nematode [40], [41], Drosophila [42] and mammals [43]. Transcriptional control of the CGL is rather complex as multiple promoters and alternative splicing are used to generate different mRNA species from both VAChT and ChAT genes [44]. Transgenic mice containing different DNA segments of the CGL fused to reporter genes have been used to identify regulatory regions that are important for the expression of VAChT and ChAT *in vivo* [19], [21], [22], [42], [45]–[47]. These studies indicate that multiple regulatory elements are necessary to control expression in the CGL and suggest that regulation of the CGL is different in different types of cholinergic neurons. Moreover, this regulatory strategy seems to be conserved in insects and vertebrates [22], [48]. A core promoter containing regulatory elements necessary to activate the CGL in cholinergic cells and to repress its activity in non-neuronal cells is present in the sequence spanning approximately 4 kb upstream of the R exon [49], [50]. Other regulatory elements have been described in the genomic region between exon-M and the first ChAT coding exon [22], however the complete set of regulatory sequences controlling the CGL remains to be determined. A cholinergic group-specific transcriptional activator has been identified in *Drosophila*. Mutant flies that lack expression of the transcription factor abnormal chemosensory jump6 (acj6) showed decreased ChAT in primary olfactory neurons, whereas expression in mechanosensory neurons was unaffected [48].

Our results give further support to the subset-specific regulation of the CGL. Because the TK-Neo cassette used to generate the VAChT^FloxNeo^ allele was introduced 450 bp upstream from the beginning of M-exon, it is reasonable to suggest that its presence interfered with the function of additional regulatory elements. As sensorymotor cholinergic neurons rely mainly on the core promoter [21], [22], VAChT expression in these neurons may be relatively preserved while all the other groups of cholinergic neurons in the brain have pronounced decrease in VAChT expression. Interestingly, ChAT expression was not altered in the VAChT^FloxNeo^ allele. This might suggest that VAChT and ChAT rely on different regulatory elements. In contrast, mice that present the VAChT^del^ allele showed an increased expression of ChAT in the spinal cord but not in the striatum. These results agree with our previous experiments showing increased ChAT expression in the spinal cord of VAChT^del^ mice [12]; see also Fig. 2 and 3). This occurs likely due to the proximity of the M-promoter of ChAT to VAChT promoters after excision of the intervening DNA sequences flanked by loxP (see Fig. 1). Our experiments suggest that whereas ChAT expression in the spinal cord may be regulated by elements that were modified by the del allele, the missing genomic fragment does not seem to be necessary for regulation of ChAT expression in the striatum (Fig. 2C and D). Overall, our experiments examining VAChT and ChAT expression point to differential gene regulation between the striatum, and likely other forebrain regions, and the spinal cord. The significance for this differential regulation for normal cholinergic physiology is poorly understood, but likely plays an important role to maintain proper expression level of these two critical cholinergic genes in these distinct sets of neurons. Although unlikely, we cannot discard the possibility that expression of the neomycin resistance protein (aminoglycoside 3′-phosphotransferase) may partially contribute to the phenotypes observed. The fact that VAChT expression was rescued by the removal of the TK-Neo cassette shows unequivocally that the two loxP sequences that flank the VAChT gene do not alter transcription.

### VAChT^FloxNeo/del^ and VAChT^FloxNeo/FloxNeo^ mice are hyperactive

As decreased VAChT expression leads to proportional decrease in ACh release in the brain [12], [13] the availability of mutant mouse lines displaying different levels of VAChT expression in the brain (VAChT^WT/del^ mice: 50% decrease; VAChT^FloxNeo/FloxNeo^ mice: 75% decrease; VAChT^FloxNeo/del^ mice: 85% decrease) provided us with unique tools to evaluate the consequences of reduced VAChT levels for brain functions. Also, understanding the consequences of decreased VAChT expression is made easier now by the new mouse lines that do not show confounding peripheral phenotypes.

Because ACh is known to play a major role in the regulation of locomotor control [51], we used these mutants to investigate the role of VAChT in locomotor activity. We found that up to 50% decrease in VAChT expression in the brain does not change locomotor activity in mice, similar to previous experiments with VAChT KD^HET^ mice and VAChT^wt/del^ mice. However, our data show clearly that a more pronounced decrease in VAChT expression causes hyperactivity in a new environment. These results suggest that release of ACh is normally required to regulate neuronal circuits controlling locomotion.

Injection of muscarinic antagonists in distinct brain regions cause pronounced augmentation in locomotor activity levels [32], [33], [35] and a hyperactivity phenotype was observed in mouse strains lacking M1 and M4 muscarinic receptors [30], [31], [52] as well as mouse strains null for the nicotinic β2 receptor [29], [36], [37]. Paradoxically, systemic injections of nicotinic agonists can cause an increase in locomotor activity [53]–[56]. However, this effect should be considered with caution, as the hyperactivity most probably results from desensitization of specific types of nicotinic receptors due to prolonged activation. Therefore, ACh may regulate locomotor circuitry in multiple and redundant ways. Our data provide additional support to the notion that insults that cause cholinergic presynaptic deficiency can also increase activity.

Locomotor hyperactivity is a symptom present in many disorders including Attention Deficit Hyperactivity Disorder (ADHD), schizophrenia, Alzheimer's diseases and some forms of autism [4]. Interestingly, all these disorders have in common some degree of cholinergic deficit. VAChT^FloxNeo/del^ and VAChT^FloxNeo/FloxNeo^ mice are novel complementary models to understand the specific consequences of decreased cholinergic activity in the brain and should be useful to further investigate the role of ACh in distinct brain functions. Importantly, as VAChT^Flox/Flox^ mice have preserved VAChT expression and do not show any phenotype, they can be used in the future to generate novel lines with suppression of ACh release in specific brain regions. These conditional mutants will be valuable to investigate the role of specific groups of cholinergic neurons in distinct brain functions.

## Materials and Methods

### Ethics Statement

The experimental procedures in this study were conducted in compliance with the Canadian Council of Animal Care (CCAC) guidelines for the care and use of animals. The protocol was approved by the University of Western Ontario Institutional Animal Care and Use Committee (protocol # 2008-089). All efforts were made to minimize the suffering of animals.

### Generation of VAChT mutant mice

Construction of the gene-targeting vector was described previously [12]. In short, one LoxP sequence was placed 260 bp upstream from the VAChT translational initiation codon, and a second LoxP was added approximately 1.5 kb downstream from the stop codon. The Neomicin-resistance gene (TK-Neo cassette) was inserted immediately after the second LoxP and was followed by a third LoxP (Figure 1). The linearized targeting vector was electroporated into J1 embryonic stem cells derived from 129/terSv mice, and selected embryonic stem cell clones harbouring homologous recombination (determined by PCR and Southern blotting (not shown) were injected into C57BL/6J blastocysts to produce chimeric mice. Germ line transmission was achieved, and mice were bred to C57BL/6J mice to produce heterozygous mutant mice (VAChT^WT/FloxNeo^). Heterozygous mice were intercrossed to generate the homozygous (VAChT^ FloxNeo/FloxNeo^) and wild-type controls (VAChT^WT/WT^) used in these experiments. VAChT^FloxNeo/del^ and VAChT^WT/del^ mice were generated by intercrossing VAChT^WT/FloxNeo^ to heterozygous VAChT KO mice (VAChT^wt/del^; [12]. Only male mice were used in this study. Animals were housed in groups of three to four per cage in a temperature-controlled room with a 12∶12 light-dark cycles in microisolator cages. Food and water were provided ad libitum. Mouse colonies were maintained at the University of Western Ontario, Canada, in accordance with Canadian Council of Animal Care (CCAC) guidelines for the care and use of animals.

### Genotyping, Southern blotting

Genotyping by PCR was performed using tail DNA as a template. The set of three primers used were P1 (5-GAGAGTACTTTGCCTGGGAG GA -3), P2 (5- GGCCACAGTAAGACCTCCCTTG -3), P3 (5- GCAAAGCTGCTATTGGCCGCTG -3) and P4 (5-TCATAGCCCCAAGTGGAGGGAGA-3). For Southern blot analysis, genomic DNA was digested with the enzymes *Bam*HI and *Sac*I. Digested DNA was subjected to electrophoresis in a 1.5% agarose gel and transferred onto a nylon membrane. After UV cross-linking, DNA on the membrane was hybridized to the NdeI/PmeI VAChT DNA fragment (see Fig. 1 for the position of the probe fragment). Detection was done using the Alkphos direct labelling and detection system kit (GE Healthcare) according to the manufacturer's instructions.

### qPCR

For real-time quantitative PCR (qPCR), total RNA was extracted using the Aurum Total RNA for fatty and fibrous tissue kit from Biorad. Quantification and quality analysis of RNA in the extracted samples was done by microfluidic analysis (Agilent Technologies' Bioanalyzer). First-strand cDNA was synthesized using the iSCRIPT cDNA SYNTHESIS KIT from Biorad. cDNA was subsequently subjected to qPCR on a CFX-96 Real Time System (Bio-Rad) using the iQ SYBR GREEN SUPERMIX (Bio-Rad). For each experiment, a non-template reaction was used as a negative control. In addition, the absence of DNA contaminants was assessed in reverse transcription-negative samples and by melting-curve analysis. Relative quantification of gene expression was done with the ΔΔ*CT* method using β-actin gene expression to normalize the data.

### Western blotting

Immunoblot analysis was carried out as described previously [12]. Antibodies used were anti-VAChT (rabbit polyclonal 1∶2000, Synaptic System, Germany), anti-CHT1 (rabbit polyclonal 1∶1000, kindly provided by R. Jane Rylett, University of Western Ontario, London, Canada), anti-CHAT (rabbit polyclonal 1∶1000, Chemicon) and anti-actin (Chemicon, CA). Images were acquired using the FluorChem Q System from Alpha Innotech and analysed using the AlphaVie software.

Immunofluorescence analysis of brain slices were performed as described previously [12]. Images were acquired using an Axiovert 200 M using the ApoTome system or a LEICA SP5 confocal microscope as previously described [17].

### Tissue ACh measurements

Brains were dissected rapidly, homogenized in 5% TCA, and centrifuged (10,000×*g* for 10 min) at 4°C. Supernatants were frozen at −80°C until use. For ACh determinations, TCA was removed with ether, and a chemiluminescent assay was done with choline oxidase as described previously [44]. The data are presented as means and standard errors of the means (SEM). One-way analysis of variance (ANOVA), followed by Bonferroni's test, was used to analyze the differences in tissue ACh concentrations in VAChT^FloxNeo/del^, VAChT^FloxNeo/FloxNeo^,VAChT^WT/del^ and wild-type controls (VAChT^WT/WT^); a *P*<0.05 was considered to be statistically significant.

### Electrophysiology

Recordings were performed on isolated hemi-diaphragm nerve-muscle preparations. Animals were euthanized and the diaphragm with attached rib bone was rapidly dissected and placed into Tyrode's solution containing NaCl (124 mM), KCl (5 mM), NaHCO_3_ (26 mM), NaH_2_PO_4_ (1.2 mM), MgCl_2_ (1.3 mM), CaCl_2_ (2.4 mM), glucose (10 mM). This solution was gassed with a mixture of 5%CO_2_/95%O_2_ and in this condition had a pH of 7.4. The diaphragm was bisected, and one half was transferred into a custom recording chamber in which the muscle was held in place with metal pins that passed through the surrounding tissue and were inserted into a Sylguard bed. During recording, the muscle was continuously perfused with gassed Tyrode solution containing 0.0003 mM tetrodotoxin to avoid spontaneous action potentials. Borosilicate (WPI) microelectrodes were fabricated on a Narashige PN-30 puller, and had resistances of 5–15 MOhm when filled with 3 M KCl. Fine branches of the motor nerve were visually identified under 100X magnification and the muscle fiber was impaled using a fine micromanipulator (WPI). Membrane potential and synaptic potentials were amplified 10X with an Axon Instruments Axoclamp 2A, and membrane potential was monitored throughout the experiment. To digitalize the miniature endplate potentials (MEPPs), the signal was high-pass filtered at 0.1 Hz to subtract the resting potential and amplified a further 200-1000X using a Cyberamp (Axon Instruments) amplifier. This signal was fed to a Lab Master A–D conversion board controlled by Strathclyde Electrophysiology Software (University of Strathclyde, Glasgow, Scotland). To measure quantal size, a software event detector was used to record 25 ms of data on either side of the MEPP. The threshold of the event detector was set just below the peak of the noise so as not to miss any small MEPPs. Under these conditions, approximately 15% of detected “MEPPs” were false positives and were manually detected and removed. To measure MEPP frequency, membrane potential was recorded without selection, and MEPPs were manually identified and counted.

### Grip force and wire-hang

Mice were brought to the testing room and allowed to acclimatize for 10 minutes before initiating tests. A Grip Strength Meter from Columbus Instruments (Columbus, OH) was used to measure forelimb grip strength as an indicator of neuromuscular function as described previously [12], [13]. Briefly, the grip strength meter was positioned horizontally and mice were held by the tail and lowered toward the apparatus. Mice were allowed to grasp the smooth, metal, triangular pull bar (forelimbs only) and were then pulled backward in the horizontal plane. The force applied to the bar at the moment the grasp was released was recorded as the peak tension (kg). The test was repeated 10 consecutive times within the same session and the highest value from the 10 trials was recorded as the grip strength for that animal. Mice were not trained prior to testing and each mouse was tested once (10 trials equal one test session).

For wire-hanging experiments the laterals of a cage top were covered with tape to prevent the mice to reach the borders [57]. The mouse was gently put on the cage top, which was then briefly shaken to induce the mouse to grasp the wire in the top. The cage top was then inverted and suspended approximately 40 cm above an empty cage. Time spent hanging upside down was determined with a cut-off time of 60 sec.

### Gait analysis

Mice were subjected to gait assessment [58] using a CatWalk automated gait analysis system (Noldus Information Technology). The apparatus is made of a 1.3 m long glass plate with dim fluorescent light beamed into the glass from the side. The reflexion of the paw in contact to the glass was recorded by a video camera. Mice were placed in the walkway and allowed free exploration for 1 min before recording the first run. A minimum of 3 correct runs (the mouse cross the walkway with no interruption or hesitation) for each mouse was recorded. Runs were analysed using the Noldus software and only the runs where it was possible to discern all steps were used for the analysis. We only used the mean stride length of hind paw as data, stride length is the distance between two successive prints of the same paw.

### Locomotor activity and habituation

Locomotor activity was automatically recorded (AccuScan Instrument, Inc. Columbus, OH). The open field arena was a 20 cm×20 cm platform surrounded by 30 cm high walls. Mice were acclimated to the testing room for 20 minutes prior to beginning the test, and had not experienced a cage change for at least 24 hours. Mice were placed in the center of the apparatus and allowed to freely explore the arena. Horizontal locomotion and rearings were recorded and used as measures of locomotion and exploration, respectively [59]. Locomotor activity was measured at 5 min intervals and cumulative counts (120 min) were taken for data analysis as described elsewhere [60]. For the intersession habituation, mice were exposed for 120 min to the same open field during 3 consecutive days. Measurements of total activity were obtained and one-way ANOVA and Tukey's Multiple Comparison Test was used to test for statistical significance. Activity was measured by the Versamax software.

### Elevated Plus-maze

Animals were placed in the center of the elevated plus maze (Med Associate Inc.) and activity was recorded for five minutes with a webcam connected to a computer. Total amount of time spent in the open and in the open sections of the maze was calculated with the Any-maze software (Stoelting Co., USA); an animal was considered to be completely within a section of the maze when its center of gravity was in this section. The result was expressed as the percentage of time spent in the open arm.

### Statistical Analysis

Data were statistically analyzed by a two-tailed Student's *t* test or by two-way or repeated measure ANOVA. If data were not normal, we used the adequate non-parametric test. The specific statistical analyses used are noted in the text and legends.

## Supporting Information

Figure S1 **Protein expression is changed in VAChT mutant mice.** a) Western blot analysis of VAChT in the striatum of VAChT^FloxNeo/FloxNeo^ mice compared to WT control and b) quantification of protein levels. c) Western blot analysis of VAChT in the striatum of VAChT^FloxNeo/Del^ mice, VAChT^WT/Del^ and VAChT^WT/WT^. d) quantification of protein levels. Actin immunoreactivity was used to correct for protein loading between experiments. Data are presented as a percentage of wild-type levels. Graphs represent average of 4–6 different mice. (*) indicates statistically different from WT/WT control (Student test, p<0.05), (**) indicates statistically different from VAChT^WT/Del^ (Student test, p<0.01).
(TIF)
