# Supplementary figures and images for: Novel Strains of Mice Deficient for the Vesicular Acetylcholine Transporter: Insights on Transcriptional Regulation and Control of Locomotor Behavior
Source: PLoS One. 2011 Mar 10;6(3):e17611. doi: 10.1371/journal.pone.0017611 (PMC3053374; doi:10.1371/journal.pone.0017611)

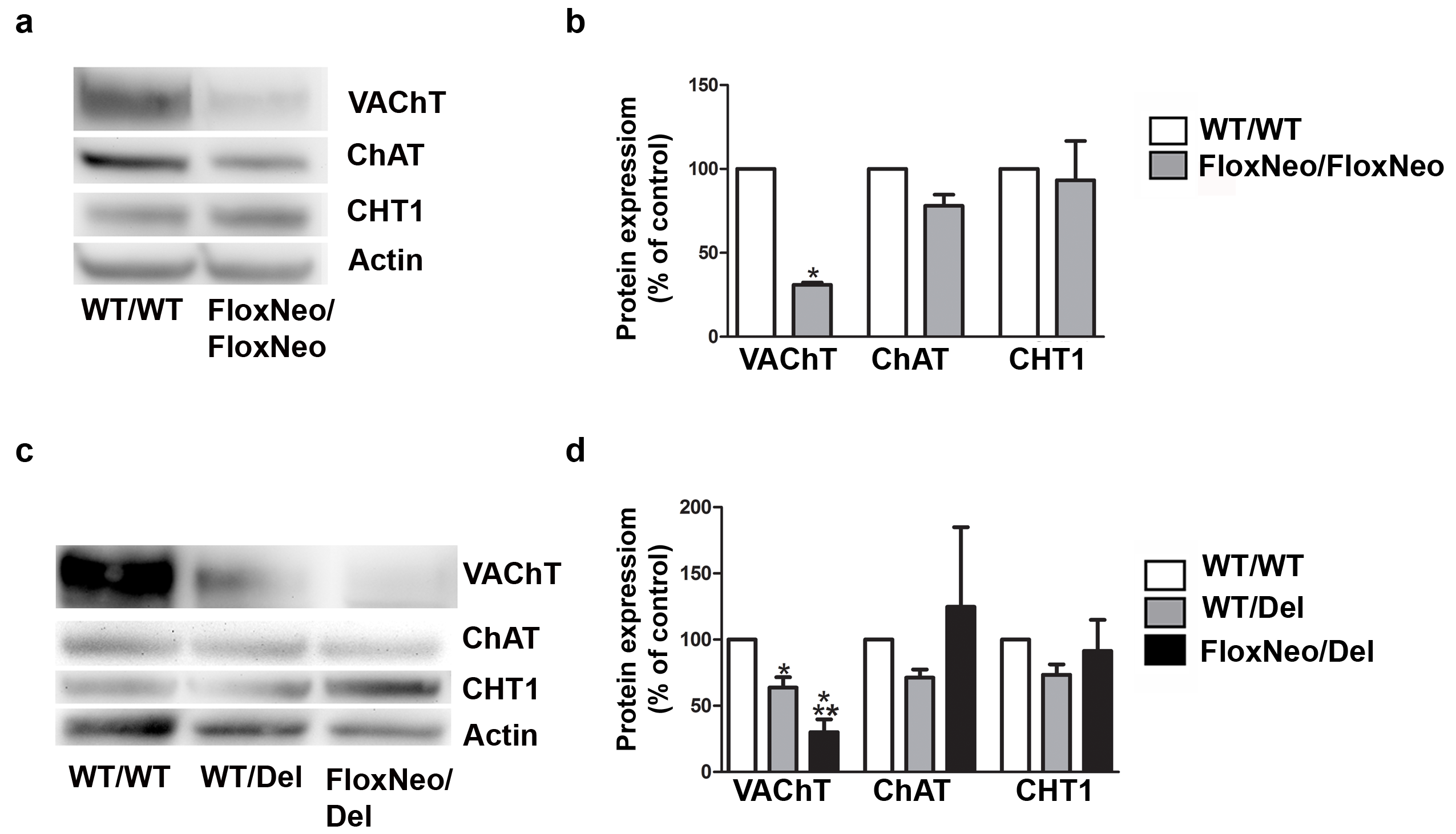

Supplement: Figure S1 — Protein expression is changed in VAChT mutant mice. a) Western blot analysis of VAChT in the striatum of VAChTFloxNeo/FloxNeo mice compared to WT control and b) quantification of protein levels. c) Western blot analysis of VAChT in the striatum of VAChTFloxNeo/Del mice, VAChTWT/Del and VAChTWT/WT. d) quantification of protein levels. Actin immunoreactivity was used to correct for protein loading between experiments. Data are presented as a percentage of wild-type levels. Graphs represent average of 4–6 different mice. (*) indicates statistically different from WT/WT control (Student test, p<0.05), (**) indicates statistically different from VAChTWT/Del (Student test, p<0.01). (TIF) [file pone.0017611.s001.tif]
